# Whole‐exome sequencing identifies a donor splice‐site variant in *SMPX* that causes rare X‐linked congenital deafness

**DOI:** 10.1002/mgg3.967

**Published:** 2019-09-03

**Authors:** Yuan Lv, Jia Gu, Hao Qiu, Huan Li, Zhitao Zhang, Shaowei Yin, Yan Mao, Lingyin Kong, Bo Liang, Hongkun Jiang, Caixia Liu

**Affiliations:** ^1^ Key Laboratory of Maternal‐Fetal Medicine of Liaoning Province Key Laboratory of Obstetrics and Gynecology of Higher Education of Liaoning Province Liaoning Centre for Prenatal Diagnosis, Research Center of China Medical University Birth Cohort Department of Gynecology & Obstetrics Shengjing Hospital Affiliated to China Medical University Shenyang Liaoning China; ^2^ Department of Otolaryngology The First Hospital Affiliated to China Medical University Shenyang Liaoning China; ^3^ Basecare Medical Device Co., Ltd. Suzhou Jiangsu China; ^4^ State Key Laboratory of Microbial Metabolism Joint International Research Laboratory of Metabolic and Developmental Sciences School of Life Sciences and Biotechnology Shanghai Jiao Tong University Shanghai China; ^5^ Department of Pediatrics The First Affiliated Hospital of China Medical University Shenyang Liaoning China

**Keywords:** DFNX4, novel variant, *SMPX*, splicing, whole‐exome sequencing, X‐linked hearing loss

## Abstract

**Background:**

X‐linked deafness‐4 (DFNX4) caused by functional loss of *SMPX* is a nonsyndromic form of progressive hearing loss with post‐lingual onset. Herein, we describe a male neonate from an ethnic Han Chinese family who presented with congenital deafness.

**Methods:**

The proband and the family members were subjected to comprehensively hearing screen. Genetic testing was carried out using whole‐exome sequencing (WES). The result was verified by Sanger sequencing. Functional characterization of the identified variant was completed by reverse transcription PCR (RT‐PCR), Sanger sequencing, and fluorogenic quantitative PCR (qPCR).

**Results:**

The proband was diagnosed with progressive sensorineural hearing loss. The proband's mother showed normal hearing at present. The proband's maternal grandmother exhibited mild HL since the age of 50. Using whole‐exome sequencing (WES), we identified a donor splice‐site variant (NM_014332.2: c.132 + 1G>A) in the *SMPX* gene in the proband. The mother and maternal grandmother were both carriers, which suggested a X‐linked inheritance of the condition in the family. RT‐PCR and Sanger sequencing revealed that four alternative splice pairs within intron 3 have led to four aberrant RNAs transcripts, including two non‐canonical splice‐pairs (GC‐AG and CT‐AG). The variant generated a novel frameshift variant, creating a premature termination codon (PTC) upstream of a newly formed splice site (p.Met45Glyfs*16). *SMPX* mRNA expression assay showed that the PTC has caused degradation of mRNA via nonsense‐mediated mRNA decay (NMD).

**Conclusion:**

This is the first study to report a *SMPX* (DFNX4) splicing variant in a Chinese family. These findings, especially congenital deafness, contributed to existing knowledge regarding the genotypic and phenotypic spectrum of *SMPX*‐associated hearing loss.

## 1 INTRODUCTION

Hearing loss (HL) is one of the most common sensory disorders, affecting over 5% of the population worldwide (466 million in total; including 432 million adults and 34 million children). It is estimated that by 2050, over 900 million people will have disabling HL (WHO data, http://www.who.int). 50%–60% of HL cases can be attributed to genetic reasons (Morton & Nance, 2006). Hereditary HL is a genetically heterogeneous disorder (Dror & Avraham, 2010) that can be divided into nonsyndromic HL (NSHL) and syndromic HL (SHL). Pathogenic variants associated with NSHL are mainly located in autosomes. To date, only six X chromosome NSHL loci and five genes have been reported, which included DFNX1 (*PRPS1,* MIM number 304500), DFNX2 (*POU3F4,* MIM number 304400), DFNX3 (unknown gene, MIM number 300030), DFNX4 (*SMPX*, MIM number 300066), DFNX5 (*AIFM1,* MIM number 300614), and DFNX6 (*COL4A6,* MIM number 300914) (https://hereditaryhearingloss.org/).

First cloned from skeletal muscle, the *SMPX* gene consists of five exons and maps to Xp.22.1 (Kemp et al., 2001; Patzak, Zhuchenko, Lee, & Wehnert, 1999). It encodes a small protein comprising 88 amino acids without no known functional domain. This gene is highly conserved across mammalian species (Palmer et al., 2001). Its variants are known to underlie X‐linked deafness‐4. The encoded protein may play a role in cytoskeletal remodeling and mechanoreception (Clark, McElhinny, Beckerle, & Gregorio, 2002), inner‐ear development and/or maintenance through interactions with various regulators such as insulin‐like growth factor 1 (IGF‐1), integrins (α8β1), and Rac1 (Cediel, Riquelme, Contreras, Diaz, & Varela‐Nieto, 2006; Grimsley‐Myers, Sipe, Geleoc, & Lu, 2009; Littlewood Evans & Muller, 2000; Palmer et al., 2001).

We hereby report on identification of a novel splicing variant (NM_014332.2:c.132 + 1G>A, p.Met45Glyfs*16) in the *SMPX* gene in a three‐generation ethnic Han Chinese family by whole‐exome sequencing (WES). Functional characterization of this variant was completed by reverse transcription PCR (RT‐PCR), Sanger sequencing, and fluorogenic quantitative PCR (qPCR).

## 2 MATERIALS AND METHODS

### 2.1 Ethical compliance

The mother of the proband has provided consent for the proband's participation in this study. Written consent was also obtained from maternal grandmother. Fetal tissue of an unborn brother carrying the same pathogenic variant was also included with ethical approval. Tissues from a voluntarily aborted fetus (with no relationship to the family) was used as the wild‐type control in accordance with the approval from the ethics committee of Shengjing Hospital of China Medical University (Ethical approval number: 2013PS33K) for the usage of patients’ blood sample, placental, and fetal tissues.

### 2.2 Patient and sample collection

**Figure 1:**
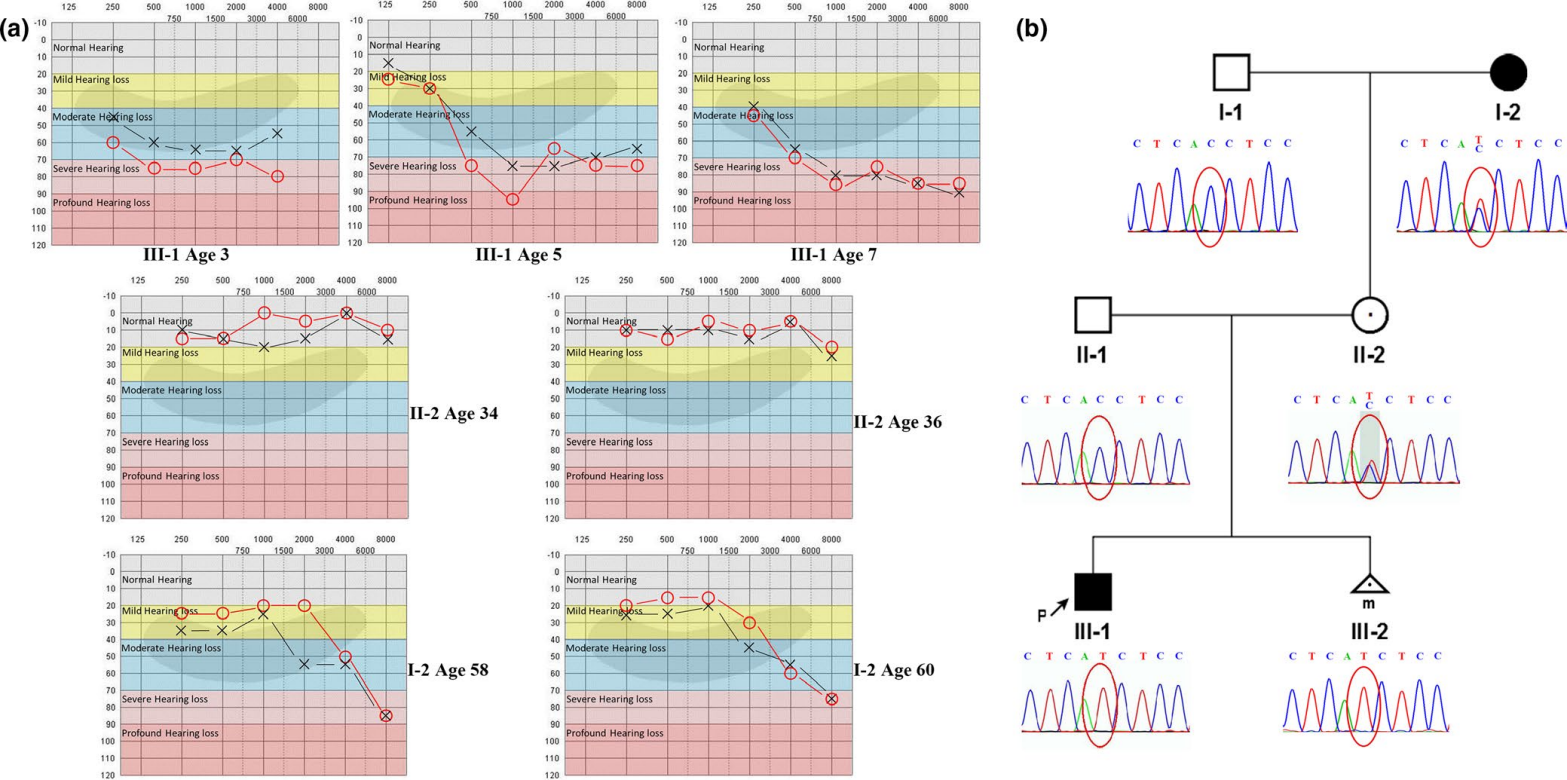
Audiograms, pedigree, and Sanger sequencing analysis. (a) Audiogram of the family member. The symbol “×” represents the left ear and “○” represents the right ear. (b) Pedigree and Sanger sequencing analysis. Affected members are indicated by filled symbols; unaffected relatives are indicated by open symbols. Heterozygous carriers are indicated with a dot in the middle of the symbol. The number of siblings is shown below the symbol. The symbol “P” and arrow indicate the proband

The three‐generation consanguineous family included 2 patients (I‐2 and III‐1) with deafness (Figure 1). The newborn hearing screening was conducted with Hearing Screening Instrument (GN OTOMETRICS A/S, Hoerskaetten 9, 2630 TAASTRUP, DENMARK) according to the standard operating procedure. Hearing assessment was conducted with Auditory Evoked Potential (GN OTOMETRICS A/S) and Pure tone Audiometry followed the standard operating procedure. After the clinical and molecular diagnosis of the proband, the mother went pregnant again. However, prenatal diagnosis indicated that the fetus carried the same pathogenic variant. After being fully informed about the result, they finally opted for abortion. Peripheral blood, tissue from a male fetus (III‐2) who carried the *SMPX* hemizygous variant and the control fetus were sampled with ethical approval.

Peripheral blood samples were taken from the family members. Genomic DNAs (gDNA) were extracted with a TIANamp Blood DNA Kit (TIANGEN). gDNA of the proband was subjected to WES. Targeted exon sequences plus flanking sequences were captured and enriched using a array‐based hybridization chip (xGen^®^ Exome Research Panel v1.0, Integrated DNA Technologies, USA) followed by HiSeq X10 (Illumina) sequencing. All variants on autosomes and sex chromosomes were annotated using the ANNOVAR tool. The pathogenicity of variants was annotated with HGMD (http://www.hgmd.cf.ac.uk/ac/index.php), Clinvar database (https://www.ncbi.nlm.nih.gov/clinvar/), and Standards and Guidelines for the Interpretation of Sequence Variants of ACMG (Richards et al., 2015). A series of in silico impact score procedures including M‐CAP (http://bejerano.stanford.edu/MCAP/), SIFT (https://sift.bii.a-star.edu.sg/), Polyphen2 (http://genetics.bwh.harvard.edu/pph2/), LRT (http://www.genetics.wustl.edu/jflab/lrt_query.html), MutationTaster (http://www.mutationtaster.org/), CADD (https://cadd.gs.washington.edu/), FATHMM (http://fathmm.biocompute.org.uk/inherited.html), PROVEAN (http://provean.jcvi.org/index.php) were used to prioritize all the variants according to ACMG guideline (PP3). The variants were filtrated using the Phenolyzer procedure (http://phenolyzer.wglab.org/) with deafness, hearing loss, and hearing impairment as the key words. Variants with minor allele frequencies < 0.01 in any of the following databases (dbSNP, ExAC, 1000 Genomes Project, gnomAD, In‐house database) were selected.

### 2.3 Sanger sequencing

*SMPX* (GenBank: NG_031916.1) variant was amplified and sequenced with a pair of *SMPX*‐specific primers: 5'‐GTTTCAGGGCTGACTGAGCA‐3′ (forward) and 5′‐ATTCCAATGGGAGCCTTTCGG‐3′ (reverse). Sanger sequencing was conducted on ABI3730 platform (Applied Biosystems).

### 2.4 Functional characterization of the splice‐site variant by RT‐PCR and QPCR

mRNA was extracted from tissue samples using a RNAprep Pure Tissue Kit (TIANGEN). Reverse transcription was conducted with a revertAid First Strand cDNA synthesis Kit (ThermoFisher Scientific, Waltham). PCR was conducted using Phanta Max Super‐Fidelity DNA Polymerase (Vazyme Biotech). Two pairs of *SMPX* primers were designed: *SMPX* Primer1 5'‐GCCAGTTTCCAATGTTAGAGCCA‐3' (forward), 5'‐CCCCCTCTGGTGAGGATTTTAGAAG‐3' (reverse); *SMPX* primer2: 5'‐TGTTAGAGCCATCCAGGCAAAT‐3' (forward), 5'‐ATCCCTCCTCAAAACCACACC‐3' (reverse). qPCR was conducted using a KAPA SYBR FAST Universal 2X qPCR Master Mix (KAPA, Massachusetts, USA) with primers designed in *SMPX* CDS sequence located upstream of the variant: 5'‐TCTCAATACCGGGAGAGGCA‐3' (forward), 5'‐TGCCTGGATGGCTCTAACAT)‐3' (reverse). Primer for GAPDH CDS, including 5'‐GAGAAGGCTGGGGCTCATTT‐3' (forward) and 5'‐AGTGATGGCATGGACTGTGG‐3' (reverse), was designed as the endogenous controls.

## 3 RESULTS

### 3.1 Clinical findings

The proband (III‐1), a six‐year‐old male, did not pass the hearing test after birth. A detailed audiological test was performed when he was three. Clinically, the proband presented moderate sensorineural HL at all frequencies with his left ear and moderate to severe HL with his right ear (Figure 1a, Table S1). Pure tone audiometry performed at the ages of 5 and 7 indicated progressive HL (Figure 1a). At the age of 7, the proband received underwent a Brainstem Auditory Evoked Potential (BAEP) test, which showed that the V wave response for his left ear was normal, but responses to I and III waves were not clear under 90 dB nHL click stimulation. Similar condition was found in his right ear under 95 dB nHL click stimulation. The V wave response thresholds of the left and the right ears were 80 dB nHL and 95 dB nHL, respectively. Above findings suggested that the proband had bilateral severe to profound HL (Figure 1a), though computer‐assisted tomography at one‐year‐old showed no abnormalities.

The proband's mother (II‐2) showed normal hearing by the BAEP test and pure tone audiometry (Figure 1a). Both of her ears’ I, III, V wave differentiations were normal under 90 dB nHL click stimulation. The V wave response thresholds of both ears were 50 dB nHL. The proband's maternal grandmother (I‐2) exhibited mild HL since the age of 50. She (I‐2) was diagnosed with slow progressive HL, including bilateral hearing impairment at high frequencies and mild HL at low frequencies (Figure 1a). Her BAEP results showed that I, III, and V wave differentiations of both ears were normal under 90 dB nHL click stimulation. The V wave response thresholds of left and right ears were 30 and 40 dB nHL, respectively. The latency and interval of each wave were within the normal range in all BAEP tests.

### 3.2 Identification of a donor splice‐site variant in *SMPX* gene

The proband (III‐1) were subjected to WES. After the data filtration according to the method described in the materials and methods, 157 variants were identified. Nine heterozygous pathogenic variants recorded in the Clinvar or HGMD data bases were identified, but none of the variants was with known deafness genes (https://hereditaryhearingloss.org/). Three of the nine variants were known to follow an autosomal recessive inheritance pattern. One variant followed autosomal dominant inheritance pattern, but the variant was reported to cause gastric cancer. According to the OMIM database (https://omim.org/), the inheritance pattern of the remaining five variants was unknown; however, none of the genes was reported to cause deafness. We then focused on variants that were absent or rare in database. Fifty‐four variants were predicted to have high to medium high functional impact according to in silico procedures. Three known deafness genes, including *SMPX*, *USH2A*, and *ILDR1*, were identified, in which the variants in *SMPX* and *USH2A* were predicted to have a high‐to‐medium high impact on the function. However, only single heterozygous variant in *USH2A* and *ILDR1* was identified, thus, the single variant cannot cause deafness in the patient. The hemizygous variant in *SMPX* (*SMPX*, NM_014332.2: c.132 + 1G>A) was finally identified as possibly being associated with deafness of the proband. This variant was annotated as pathogenic according to ACMG guidelines, which was accord with PVS1, PM2, and PP3. No other deleterious variants in any other genes were identified to cause deafness of the patient. Sanger sequencing confirmed that the mother (II‐2) and maternal grandmother (I‐2) were carriers and his unborn brother was hemizygous variant (Figure 1b).

### 3.3 Functional characterization of the donor splice‐site variant in the *SMPX* gene

**Figure 2:**
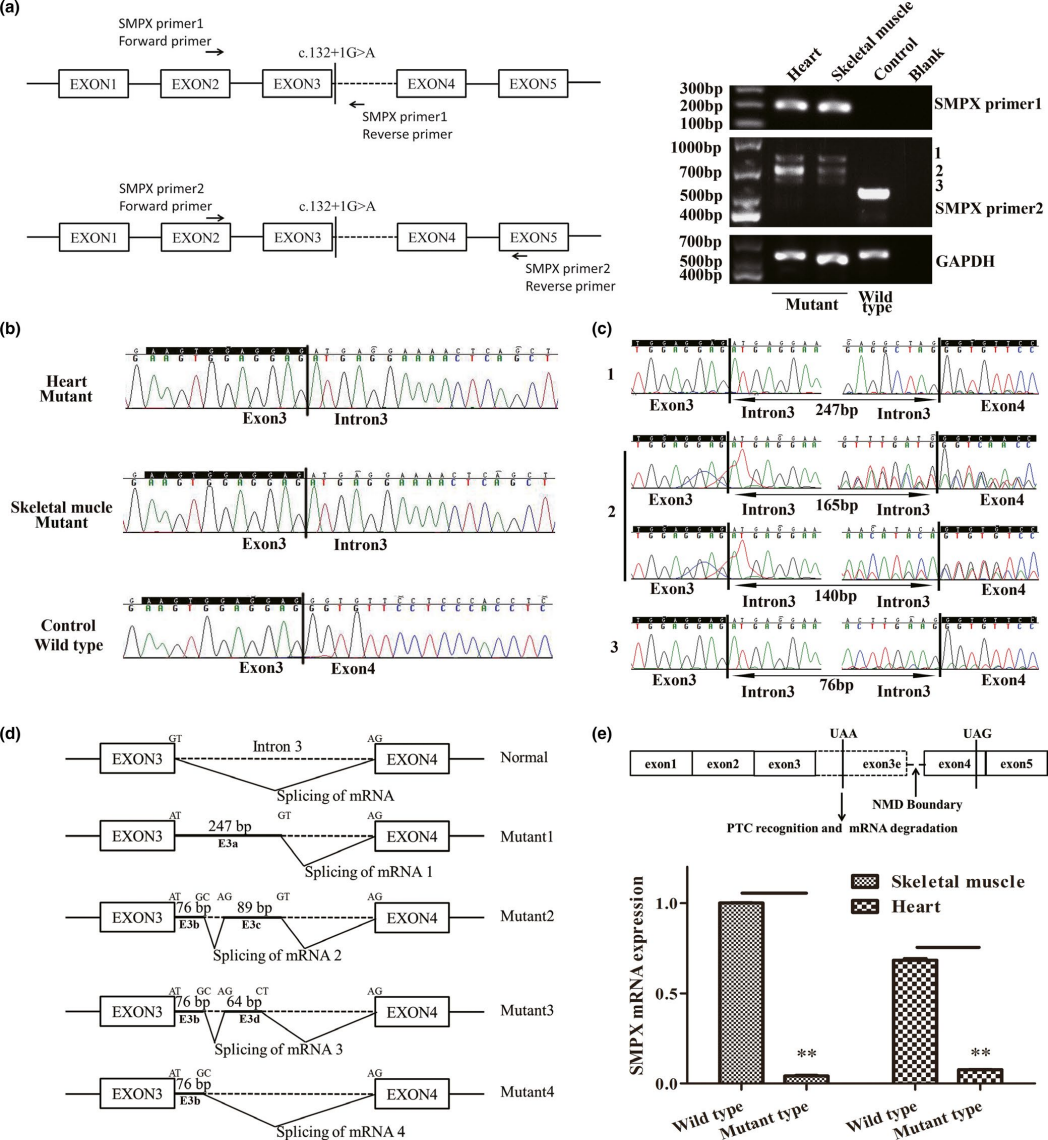
Functional characterization of the donor splice‐site mutation in the SMPX gene. (a) Primer design for RT‐PCR and agarose gel electrophoresis of PCR products with mutant (III‐2) and control tissues. (b) Sanger sequencing of heart and skeletal muscle (III‐2) with primer 1 and control skeletal muscle with primer 2. (c) Sanger sequencing of the PCR products labeled as 1, 2, and 3 in (a). (d) The new forms of splicing due to the splice‐site mutation. (e) Location of PTC and SMPX mRNA expression assay by qPCR

To confirm whether the variant altered splicing, in vivo functional characterization of the variant was conducted. RT‐PCR using *SMPX*‐primer1 produced a product with heart and skeletal muscle mRNA, while no product was obtained with the control mRNA, demonstrating that this variant leads to a new transcript including intron 3, as confirmed by Sanger sequencing (Figure 2b). RT‐PCR using control mRNA and *SMPX*‐primer2 produced a normal fragment, but mRNA from mutant‐type fetal tissue showed three extended fragments (Figure 2a). The fragments were cut from the agarose gel and Sanger sequencing was performed, four different erroneous transcript isoforms were found (Figure 2c and d). Two types of aberrant RNAs transcripts were clearly identified by agarose gel recovery and Sanger sequencing (Figure 2c1 and c3). When Sanger sequencing was conducted with band 2 in Figure 2a, ambiguous peaks were obtained, indicating two or more aberrant RNAs transcripts. After unscrambling the sequences with ambiguous peaks, two aberrant RNAs transcripts were identified (Figure 2c2). The newly formed splice junctions can be found in Table 1. Non‐canonical splice junction of GC‐AG pair and CT‐AG pair were identified (Figure 2d).

**Table 1:** Splicing patterns of different transcript isoforms and HSF consensus value of the potential splice‐site

Mutation	Splicing pattern	HSF consensus value	
		Donor	Acceptor
Wild type	AGTGGAGGAG 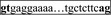 GGTGTTCCTC	95.15	93.97
Mutant 1	TTACTTGAAG 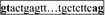 GGTGTTCCTC	75.99	93.97
Mutant 2	CAGAGGCTAG 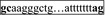 TTGTCTTGCT	69.25	77.29
	TTACTTGAAG 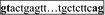 GGTGTTCCTC	75.99	93.97
Mutant 3	CAGAGGCTAG 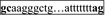 TTGTCTTGCT	69.25	77.29
	AATTTAAGTA 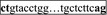 GGTGTTCCTC	NA	93.97
Mutant 4	CAGAGGCTAG 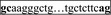 GGTGTTCCTC	69.25	93.97

Note

Exon sequences in wild‐type and mutant tissues are shown in capital letters, with intron sequences in lower case. Acceptor and donor ends are shown in bold, and the corresponding sequence being spliced out is boxed.

All four aberrant splice junctions predicted a frameshift in the ORF. Thus, premature termination codon (PTC) will result in truncation of protein to 60 amino acids (p.Met45Glyfs*16) (DNAMAN software, https://www.lynnon.com/pc/framepc.html). mRNA expression was detected through qPCR. The mRNA expression was 4.8% and 6.6% in skeletal muscle and heart compared with control, respectively, indicating that mutant mRNA was degraded via nonsense‐mediated mRNA decay (NMD) (Figure 2e).

## 4 DISCUSSION

Nine pathogenic *SMPX* variants have been associated with X‐linked NSHL to date. Of note, all such variants were predicted to result in truncated proteins or frameshift with a PTC (Table 2) (Abdelfatah et al., 2013; Deng et al., 2018; Gao et al., 2018; Huebner et al., 2011; Niu et al., 2017, 2018; Schraders et al., 2011). Three of such variants were of nonsense type, which were predicted to introduce a PTC and result in a truncated protein (Table 2) (Huebner et al., 2011; Schraders et al., 2011). Five variants were predicted to cause a frameshift and a PTC, which may generate a truncated protein (Table 2) (Abdelfatah et al., 2013; Deng et al., 2018; Gao et al., 2018; Niu et al., 2017; Schraders et al., 2011). The remaining one, a splicing variant, was predicted to result in an aberrant RNAs transcript and cause a frameshift and a PTC (Table 2) (Niu et al., 2018).

**Table 2:** Pathogenic variants identified in *SMPX* gene and the associated deafness

cDNA	Amino Acid	Function	Gender	Age of onset	Severity	Progressive	Reference
c.109G > T	p.Glu37*	Predicted to introduce a PTC and result in a truncated protein; Predicted to undergo NMD	M	3–7	Moderate to Profound	Yes	(Huebner et al., 2011)
			F	Second to third decades	Bilateral; Moderate to Severe hearing loss after 10–15 years	Yes	
c.175G > T	p.Gly59*		M	5–7	Bilateral; Moderate to severe or profound	Yes	
			F	Fourth decades	Bilateral; Moderate	Yes	
c.130delG	p.Glu44Argfs*37	Predicted to leads to a frameshift and a PTC;	M	4	Bilateral; Severe	Yes	(Schraders et al., 2011)
c.214G > T	p.Glu72*	Predicted to introduce a PTC and result in a truncated protein	M	2–10	Bilateral; Moderate to Profound	Yes	
			F	3–48	Unilateral or Bilateral; Mild to Profound	Yes	
c.99delC	p.Arg34Glufs*47	Predicted to cause a frameshift and a PTC;	M	First decade	Bilateral; Severe	Yes	(Abdelfatah et al., 2013)
		May not be degraded via NMD	F	4–62	Unilateral or Bilateral; Variable	Yes	
c.217dupA	p.Ile73Asnfs*5	Predicted to cause a frameshift and a PTC;	M	0–8	Bilateral; Severe to Profound	Yes	(Niu et al., 2017)
		Predicted immune to NMD	F	Third to fourth decades	Unilateral or Bilateral; Mild	NA	
c.87dupA	p.Gly30Argfs*12	Predicted to cause a frameshift and a PTC;	M	7	Bilateral; Severe	Yes	(Deng et al., 2018)
			F	>30	Unilateral or Bilateral; Severe	Yes	
c.133−1G > A	p.Gly45Valfs*36	Predicted to cause a frameshift and a PTC;	M	Childhood	Bilateral; Profound	Yes	(Niu et al., 2018)
			F	Childhood	Unilateral; Severe	Yes	
c.29insA	p.Asn10Lysfs*3	Predicted to cause a frameshift, premature truncation protein	M	5–10	Bilateral; Profound	Yes	(Gao et al., 2018)
			F	Second to third decades	Bilateral; Moderate to profound; Symmetric or asymmetric	Yes	
c.132 + 1G>A	p.Met45Glyfs*16	Identified four aberrant RNAs transcripts; Predicted to cause a frameshift and a PTC;	M	Newborn	Bilateral; Severe to profound	Yes	This study
		mRNA decay via NMD	F	>50	Bilateral; Moderate to severe at high frequencies; Mild at low frequencies	NA	

Note

NMD, Nonsense‐mediated mRNA decay; M, Male; Fm, Female.

In this study, we have identified a novel splicing variant (NM_014332.2: c.132 + 1G>A, p.Met45Glyfs*16). All of the four aberrant RNAs transcripts have resulted in a PTC upstream of newly formed splice‐sites.

HL patients with *SMPX* variants were first identified by Huebner et al. (2011) and Schraders et al. (2011). Together they have identified four deafness families from Germany, Spain and Netherlands. The affected males all presented progressive HL with early onset, initial HL at high frequencies, and moderate to profound deafness; while the affected females all presented progressive HL with later onset from twenties to forties, and moderate to profound deafness (Table 2). In 2013, Abdelfatah, et al. found two Newfoundland deafness families caused by *SMPX* variant, in which the male patients show HL in the first decade of life and flat moderate HL by age 2. The females’ phenotypes were highly variable, including unilateral or bilateral, early or late onset, steeply sloping, and asymmetrical or symmetrical hearing impairment (Abdelfatah et al., 2013). In addition, the author speculated that although some male patients passed the newborn hearing screening test, they still could have had a non‐detectable mild HL when screened (Table 2) (Abdelfatah et al., 2013). Also, Niu et al. identified two families with pathogenic *SMPX* variants (Niu et al., 2017, 2018). The phenotypes of the patients were similar to the previously reported ones, the early onset age (0–8 years) was observed in affected males, and one male patient failed the newborn hearing screen. But other males were still suspected to have non‐detectable mild HL at the time of birth, even though they passed the newborn screen. The authors speculated that these patients could not be excluded from congenital deafness (Niu et al., 2017, 2018). Furthermore, Deng et al. and Gao et al. recently identified two HL families, in which the onset of male patients were 5–10 years old, while the hearing impairment of females arose at second to third decades (Table 2) (Deng et al., 2018; Gao et al., 2018). Taken together, most of the male patients passed the newborn hearing screening, and their hearing impairment was first noticed between 2 to 10. For female patients, their phenotypes are milder and the age of onset is highly varied (3–62 years old). The cause for this gender difference is unclear, we speculate it may due to X‐inactivation (Gao et al., 2018; Lyon, 1961; Petersen, Wang, & Willems, 2008), for which further research is required.

In our study, the proband exhibited a deafness phenotype during neonatal period, which is different from the recognized postlingual onset caused by functional loss of *SMPX*. Combining the speculation of Niu et al. (2017), we propose that pathogenic variants in *SMPX* may also lead to congenital deafness at least in the Chinese population. The variant was inherited from his mother (II‐2) and maternal grandmother (I‐2), despite that his mother exhibited no hearing impairment, while his maternal grandmother only exhibited mild HL. Furthermore, based on our clinical observations, we could not distinguish the high frequencies HL from presbycusis. For most people, the first sign of presbycusis is the loss of threshold sensitivity in the high frequency at the age of 60 (Gates & Mills, 2005). Collectively, with the pathogenic variant and the age of onset taken into consideration, the HL of the maternal grandmother may partly result from the molecular etiology. However, further follow‐up surveys are needed to confirm our speculation. Except for congenital deafness, all other phenotypes of our proband's family are similar to those in previous reports, including sensorineural HL, late onset in females, and initial HL at high frequencies (Deng et al., 2018; Gao et al., 2018; Schraders et al., 2011; Stanton et al., 2014; Weegerink et al., 2011).

The variant captured our attention because that it not only formed four different aberrant RNAs transcripts, but also gained two non‐canonical splice pairs (GC‐AG, CT‐AG). In mammalian genomes, 99.24% of splice‐site pairs comply to the GT‐AG rule, 0.69% ~ 0.9% to GC‐AG and 0.05% ~ 0.09% to AT‐AC, with only 0.02% consisting of other types of non‐canonical splice sites (Burset, Seledtsov, & Solovyev, 2000, 2001; Sheth et al., 2006). Compared to GC‐AG rule, CT‐AG splice pair is an even more rare non‐canonical splice‐site, with only six EST‐supported pairs showing this splicing pattern (Burset et al., 2000). We were able to predict the three newly formed aberrant RNAs transcripts, including GT‐AG and GC‐AG with a HSF consensus value ranging from 69.25 to 93.97 (Table 1). None of splicing prediction software was able to find the CT‐AG splice pair. The reason that so many cryptic products were formed may be due to the disruption of the most efficient wild‐type GT‐AG splice pair and activation of closely graded U2‐type splice pairs.

The newly formed PTC was within the extended exon 3e, which may result in NMD (Figure 2e). Although the nine known pathogenic variants may all result in truncated proteins or frameshift with a PTC. There has been no evidence suggesting that these variants may result in mRNA decay via NMD at the mRNA level (Abdelfatah et al., 2013; Huebner et al., 2011; Niu et al., 2017), which may be attributed to tissue‐specific expression and difficulties with tissue acquisition. Although in vivo functional study was conducted with the mutant type fetus tissue other than the proband sample (low *SMPX* mRNA expression in peripheral blood), we suspected that, with the same genetic basis, the variant may give rise to similar aberrant RNAs transcripts and mRNA degradation pathway.

In conclusion, we have identified a novel *SMPX* splicing variant (NM_014332.2: c.132 + 1G>A, p.Met45Glyfs*16) in a Chinese family. The variant may cause aberrant splicing of intron 3 and form four aberrant RNAs transcripts. The PTC upstream of the aberrant splicing sites may result in NMD. Taken together, the variant of *SMPX* may lead to congenital deafness in males and late onset deafness in females. Our result has expanded the spectrum of *SMPX*‐associated HL and facilitate genetic counseling for the affected family.

## CONFLICT OF INTEREST

The authors declare no conflicts of interest.
