# Supplementary material for: Whole‐exome sequencing identifies a donor splice‐site variant in SMPX that causes rare X‐linked congenital deafness
Source: Mol Genet Genomic Med. 2019 Sep 3;7(11):e967. doi: 10.1002/mgg3.967 (PMC6825843; doi:10.1002/mgg3.967)
Supplement: Supplementary file 1 [file MGG3-7-e967-s001.docx]

Supplementary Table1. Audiological examination of the proband at three years old

| **Acoustic immittance** | | | | | | | | |
| --- | --- | --- | --- | --- | --- | --- | --- | --- |
| Ear | 1000Hz Tympanic curve | 226Hz Tympanic curve | | Tympanic pressure daPa | | | compliance value (ml) | |
| Left | Negative single peak | C | | -160 | | | 0.2 | |
| Right | No peak | C | | -125 | | | 0.2 | |
| **Latency of auditory brainstem response** | | | | | | | | |
| Acoustic stimulation | Intensity dBnHL | Ⅰ(ms) | Ⅲ(ms) | Ⅴ(ms) | Ⅰ-Ⅲ(ms) | Ⅲ-Ⅴ(ms) | | Ⅰ-Ⅴ(ms) |
| Left | 100 | 1.48 | 3.85 | 5.78 | 2.37 | 1.93 | | 4.30 |
| Right | 100 | 1.58 | 3.95 | 5.95 | 2.37 | 2.00 | | 4.37 |
| **Auditory brainstem response threshold** | | | | | | | | |
| Left air conduction threshold | 60 dBnHL | | | | | | | |
| Right air conduction threshold | 75 dBnHL | | | | | | | |
| **40Hz AERP** Acoustic stimulation: Tone burst Status: Drug sleep Frequency: 1000Hz | | | | | | | | |
| Left threshold | 70 dBnHL | | | | | | | |
| Right threshold | 80 dBnHL | | | | | | | |
| **DPOAE** Two pure tone frequency ratio f2/f1=1.22 L1=65 dBSPL L2=55 dBSPL | | | | | | | | |
| Left | Not Pass | | | | | | | |
| Right | Not Pass | | | | | | | |
